# DLL3-TCE治疗SCLC中国专家共识

**DOI:** 10.3779/j.issn.1009-3419.2026.106.10

**Published:** 2026-04-20

**Authors:** 中国临床肿瘤学会小细胞肺癌专家委员会

**Keywords:** 肺肿瘤, δ样配体3, T细胞衔接器, 塔拉妥单抗, 专家共识, Lung neoplasms, Delta-like ligand 3, T cell engager, Tarlatamab, Expert consensus

## Abstract

小细胞肺癌（small cell lung cancer, SCLC）因其侵袭性强、早期转移率高、预后差，长期以来是肺癌治疗的难点。近年来，免疫检查点抑制剂（immune checkpoint inhibitors, ICIs）的出现显著提升了SCLC患者的总生存，成为SCLC治疗新标准，然而患者整体预后仍有较大提升空间。δ样配体3-T细胞衔接器（delta-like ligand 3-T cell engager, DLL3-TCE）作为一种新兴的靶向T细胞的免疫疗法，可精准地引导T细胞攻击肿瘤细胞，在SCLC治疗中展现出突破性潜力。塔拉妥单抗作为其中代表性药物目前已获批用于临床，多款TCE药物正在临床开发中。尽管DLL3-TCE药物在SCLC临床应用中展现出良好前景，但其作为新兴治疗手段，广大临床医生对该类药物的作用机制、适用人群及临床应用管理尚缺乏深入了解。为规范DLL3-TCE在中国的临床应用，中国临床肿瘤学会小细胞肺癌专家委员会组织共识组专家基于循证医学证据与专家实践经验，制定了《DLL3-TCE治疗SCLC中国专家共识》，旨在为DLL3-TCE类药物的临床应用提供规范化指导。

## Abstract (en)

Small cell lung cancer (SCLC), characterized by high aggressiveness, early metastasis, and poor prognosis, has long been a major challenge in lung cancer treatment. In recent years, the advent of immune checkpoint inhibitors (ICIs) has significantly improved overall survival in patients with SCLC and has become a new standard of care; however, substantial room for further improvement in clinical outcomes remains. As an emerging T cell-targeted immunotherapy, delta-like ligand 3-T cell engager (DLL3-TCE) precisely directs T cells to attack tumor cells and have demonstrated breakthrough potential in the treatment of SCLC. Tarlatamab, a representative agent in this class, has been approved for clinical use, and multiple TCE agents are currently under clinical development. Although DLL3-TCE shows promising prospects in the clinical management of SCLC, as a novel therapeutic modality, many clinicians still lack an in-depth understanding of their mechanisms of action, appropriate patient populations, and clinical management. To standardize the clinical application of DLL3-TCE in China, the Small Cell Lung Cancer Expert Committee of the Chinese Society of Clinical Oncology convened a consensus panel to develop the Chinese Expert Consensus on Delta-like Ligand 3-T Cell Engager Therapy for SCLC based on evidence-based medicine and expert practical experience, with the aim of providing standardized guidance for the clinical use of DLL3-TCE agents.

肺癌是全球发病率和死亡率最高的恶性肿瘤之一。小细胞肺癌（small cell lung cancer, SCLC）为高侵袭性肿瘤，占所有肺癌病例的10%-15%，具有增殖迅速、侵袭性强、早期转移等特征。约70%的患者初诊时已为广泛期SCLC（extensive-stage SCLC, ES-SCLC），预后不佳，2年总生存（overall survival, OS）率仅为8%^[1]^。既往30年间以铂类为基础的化疗方案一直是SCLC的主要治疗手段，虽初始应答率高，但多数患者出现快速复发和耐药，中位OS仅为8-10个月^[2]^。近年来，免疫检查点抑制剂（immune checkpoint inhibitors, ICIs）的出现显著改善了SCLC患者的生存获益，以程序性死亡受体1/程序性死亡配体1（programmed death protein-1/programmed death ligand-1, PD-1/PD-L1）抑制剂为代表的免疫治疗联合化疗，已成为ES-SCLC的一线标准治疗方案^[3]^。然而，部分患者仍面临疾病快速进展的风险，且一线化免治疗进展后，后线治疗选择非常有限^[2,4,5]^，亟需探索新的治疗策略，以进一步改善SCLC患者的整体预后。

T细胞衔接器（T cell engager, TCE）是一类靶向T细胞的新型免疫疗法，首款靶向CD19/CD3的TCE药物于2014年获批用于前体B细胞急性淋巴细胞白血病的治疗，截至目前多款靶向CD19/CD3、CD20/CD3、BCMA/CD3与GPRC5D/CD3等靶点的TCE药物相继获批用于血液肿瘤的治疗^[6]^。而在实体瘤领域，由于抗原表达异质性、肿瘤免疫微环境复杂性等因素的限制，TCE药物的研发面对诸多挑战，直到首个靶向δ样配体3（delta-like ligand 3, DLL3）/CD3的TCE药物塔拉妥单抗（Tarlatamab）在SCLC中取得成功，标志着实体瘤TCE治疗时代正式开启^[7,8]^。DLL3是Notch信号通路中的一种抑制性配体^[9]^，在健康细胞中极少表达，而在神经内分泌肿瘤（neuroendocrine cancer, NEC）尤其是SCLC细胞表面特异性高表达，因此被视为SCLC治疗的理想靶点。靶向DLL3的TCE（DLL3-TCE）药物，以塔拉妥单抗为代表，在SCLC治疗领域展现出突破性的潜力，开创了SCLC治疗的新时代，为患者带来了全新治疗选择^[8]^。

尽管DLL3-TCE药物在SCLC临床应用中展现出良好前景，但作为新兴治疗手段，广大临床医生对其作用机制、临床应用、适用人群缺乏深入的了解；此外，由于TCE药物独特的作用机制，其治疗可能会出现一些区别于传统化疗和免疫治疗的不良事件（adverse events, AEs），包括细胞因子释放综合征（cytokine release syndrome, CRS）和免疫效应细胞相关神经毒性综合征（immune effector cell-associated neurotoxicity syndrome, ICANS），广大临床医生尚缺乏对CRS和ICANS发生特征、早期用药与监测方案及AEs处理的系统性认识。基于此，中国临床肿瘤学会（Chinese Society of Clinical Oncology, CSCO）小细胞肺癌专家委员会组织专家组，依据最新的循证医学证据并结合中国专家的临床实践经验，制定《DLL3-TCE治疗SCLC中国专家共识》。共识主要聚焦DLL3-TCE的临床应用和AEs管理两大部分，旨在推动DLL3-TCE药物在SCLC治疗中的规范化应用，从而进一步改善中国SCLC患者的生存。

## 1 共识制定方法学

### 1.1 专家组成员

本共识由CSCO小细胞肺癌专家委员会组织50名肿瘤内科、放疗科、呼吸科、血液科多学科专家共同编写。专家组基于现有的国内外循证医学证据和临床经验，拟定共识的框架、关键临床问题，并于共识研讨会进行充分的讨论。

### 1.2 文献检索

本共识文献检索数据库包括PubMed和Embase，检索时限自2010年1月1日至2025年12月11日，临床应用部分使用检索式（#1 OR #2）AND #3，安全性管理部分使用检索式（#1 OR #2）AND #4（表1）。文献纳入类型包括随机对照试验、系统综述、荟萃分析、回顾性研究、队列研究、临床病例系列研究和指南。通过阅读文献摘要筛选与具体临床问题有关的文献，将支持共识形成的重要文献证据整理到共识证据与考量部分。

**表1:** 文献检索关键词

分类	关键词
1#TCE	T cell engager OR T cell engagers OR Bispecific T-cell engager OR Bispecific T-cell engagers OR Trispecific T-cell engager OR Trispecific T-cell engagers
2#DLL3	DLL3 OR DLL-3 OR Delta like canonical Notch ligand 3 OR Tarlatamab OR AMG 757 OR AMG757 OR Gocatamig OR DS 3280 OR DS3280 OR HPN 328 OR HPN 823 OR HPN328 OR HPN823 OR MK 6070 OR MK6070 OR 225Ac-ETN 029 OR 225Ac-ETN029 OR SYS 6040 OR SYS6040 OR IBI 3009 OR IBI3009 OR RG 6810 OR RG6810 OR BHP 01 OR BHP01 OR FZ AD005 OR FZ-AD-005 OR FZAD005 OR ABD 147 OR ABD147 OR SNC 115 OR SNC115 OR ALPS 12 OR ALPS12 OR RG 6524 OR RG6524 OR RO 7616789 OR RO7616789 OR LB 2102 OR LB2102 OR NEUK200 13 OR NEUK20013 OR AB 248 OR AB248 OR Obrixtamig OR BI 764532 OR BI764532 OR OBT 620 OR OBT620 OR 4SCAR DLL3 T cell OR 4SCAR DLL3 T cells OR IDE 849 OR IDE849 OR SHR 4849 OR SHR4849 OR Alveltamig OR ZG 006 OR ZG006 OR Peluntamig OR PT 217 OR PT217 OR Zocilurtatug pelitecan OR YL 212 OR YL212 OR ZL 1310 OR ZL1310
3#SCLC	Small cell lung cancer OR Small cell lung carcinoma OR Small cell lung tumor OR Small cell lung tumour OR Small cell pulmonary cancer OR Small cell pulmonary carcinoma OR Small cell pulmonary tumor OR Small cell pulmonary tumour OR SCLC OR Limited stage small cell lung cancer OR Limited-stage small cell lung cancer OR Limited stage small cell lung carcinoma OR Limited-stage small cell lung carcinoma OR Extensive stage small cell lung cancer OR Extensive stage small cell lung carcinoma OR Extensive-stage small cell lung cancer OR Extensive-stage small cell lung carcinoma
4#AE (CRS/ICANS)	Cytokine release syndrome OR Cytokine storm OR Immune effector cell-associated neurotoxicity syndrome OR Immune effector cell neurotoxicity OR Immune therapy neurotoxicity OR CAR-T therapy neurotoxicity OR Immune effector cell central nervous system toxicity OR Immune effector cell neuropsychiatric symptoms OR Immune effector cell blood-brain barrier disruption OR Immune effector cell inflammatory cytokines neurotoxicity OR Immune effector cell microglial neurotoxicity

CRS：细胞因子释放综合征；DLL3：δ样配体3；ICANS：免疫效应细胞相关神经毒性综合征；SCLC：小细胞肺癌；TCE：T细胞衔接器；CAR-T：嵌合抗原受体T细胞免疫疗法。

### 1.3 形成共识的方法学

#### 1.3.1 证据等级评定标准和推荐强度

本共识的推荐意见按证据级别和推荐强度进行分别评估。循证医学证据级别分为高、稍低和低3个等级。对于初拟的共识推荐意见，结合专家组广泛认可的临床经验和投票一致性，形成推荐等级，推荐强度根据专家投票分为强、中、弱3个等级（表2）。

**表2:** 证据级别与推荐强度

项目		详细说明
证据级别	高（1类）	严谨的meta分析、大型随机对照研究
	稍低（2类）	一般质量的meta分析、小型随机对照研究、设计良好的大型回顾性研究、病例-对照研究
	低（3类）	非对照的单臂临床研究、病例报告、临床前研究、专家观点
推荐强度	强	绝大多数专家达成共识（90%-100%一致）
	中	多数专家达成共识但少数专家存在分歧（60%-89%一致）
	弱	专家未达成一致性结论（<60%一致）

#### 1.3.2 关键临床问题遴选及推荐意见形成过程

共识专家组从临床实践治疗需求出发，基于多轮讨论共同遴选和确定拟纳入的关键临床问题。基于关键临床问题，梳理相关循证医学证据，根据证据级别分类及相关性，讨论决定拟纳入的证据，并于共识讨论会进行充分讨论，收集专家组针对拟纳入的关键临床问题和相关支持性证据的建议，由执笔专家汇总专家组意见后，确定最终纳入的临床问题、支持性证据及共识框架。

基于确定的临床问题和支持性证据，由执笔专家组织初稿撰写，通过专家组在线调研问卷及专家讨论会反馈意见和投票，形成最终的推荐意见和推荐等级，并形成共识定稿。本共识已在国际实践指南注册与透明化平台（Practice guideline REgistration for transPAREncy, PREPARE）上注册，注册号为PREPARE-2026CN396。

## 2 DLL3-TCE药物概述

### 2.1 TCE药物的作用机制

#### 2.1.1 TCE的分子结构和功能

TCE是一类双特异性或多特异性抗体，其典型分子结构由两个单链可变区片段（single-chain variable fragments, scFv）通过柔性肽链连接而成，一端特异性识别肿瘤细胞表面的肿瘤相关抗原（tumor-associated antigen, TAA），如BCMA、CD19、DLL3等；另一端则以高亲和力结合T细胞表面的CD3分子，从而衔接T细胞和肿瘤细胞，精准地将T细胞定向募集至肿瘤部位并诱导其激活并杀伤肿瘤细胞^[4,10,11]^。在血液肿瘤领域，靶向CD19、CD20、BCMA等的TCE药物已广泛用于淋巴瘤和多发性骨髓瘤的治疗^[6]^。在实体瘤领域，靶向DLL3的TCE在复发/难治性SCLC中取得的突破性成果，标志着此类疗法在实体瘤领域的首次重大成功^[8]^。目前针对其他实体瘤靶点（如Claudin18.2、PSMA、HER2等）的TCE正处于广泛的临床研究阶段（表3），展现了广阔的探索前景^[12]^。

**表3:** TCE药物在实体瘤领域的发展概况

治疗领域	药物名称	药物靶点	研发进度
SCLC/NEC	塔拉妥单抗	CD3×DLL3	已获批ES-SCLC二线治疗适应证（FDA）以及ES-SCLC三线及以上治疗适应证（FDA和NMPA）；一线ES-SCLC及LS-SCLC III期临床研究进行中
	Obrixtamig (BI 764532)	CD3×DLL3	III期临床研究
	Alveltamig (ZG006)	CD3×DLL3×DLL3	III期临床研究
	Gocatamig (MK-6070/HPN328)	CD3×DLL3×albumin	II期临床研究
	SHR-7787	CD3×DLL3	II期临床研究
	HLX-3901	CD3×DLL3×DLL3×CD28	I期临床研究
	RG6524 (RO7616789/Clesitamig)	CD3×DLL3×CD137	I期临床研究
前列腺癌	AZD6621	CD3×CD8×STEAP2	I/II期临床研究
	JANX007	CD3×PSMA	I期临床研究
	Xaluritamig (AMG 509)	CD3×STEAP1	I期临床研究
胰腺癌	QLS31905	CD3×Claudin18.2	III期临床研究
结直肠癌	MGD007	CD3×gpA33	I期临床研究
胶质母细胞瘤	BRiTE (APTN-101)	CD3×hEGFRvIII	I期临床研究
HER2^+^乳腺癌	Runimotamab (BTRC 4017A)	CD3×HER2	I期临床研究

ES-SCLC：广泛期小细胞肺癌；LS-SCLC：局限期小细胞肺癌；NEC：神经内分泌肿瘤；HER2：人表皮生长因子受体2；FDA：美国食品药品监督管理局；NMPA：中国国家药品监督管理局。

基于分子结构，TCE可大致分为含Fc结构域与不含Fc结构域两类。早期的TCE仅由两个scFv组成，是一种非IgG样抗体，分子量小，组织穿透能力强，但半衰期较短，需要持续静脉输注以维持治疗血清浓度^[10]^。为了克服这一限制，半衰期延长的TCE（half-life extended TCE, HLE-TCE）通过在TCE分子中添加Fc结构域设计而成，以延长患者的给药间隔。同时，为了避免TCE药物的Fc段与免疫细胞上的Fc受体之间的相互作用，如抗体依赖的细胞介导的细胞毒性，通过结构改造来消除Fc结构域与Fc受体的结合，开发出了具有静默Fc结构域的抗体（如塔拉妥单抗），以减少或避免不必要的效应功能，同时保留延长半衰期的能力，保持对治疗药物的稳定暴露^[13]^。

#### 2.1.2 肿瘤免疫逃逸与TCE的作用机制

正常免疫应答过程中，肿瘤细胞上的主要组织相容性复合体（major histocompatibility complex, MHC）分子呈递TAAs，与T细胞受体（T cell receptor, TCR）相互作用，从而诱导T细胞激活以消除肿瘤细胞^[14]^。而肿瘤细胞可通过多种机制逃避免疫监视，包括下调MHC I类分子的表达，限制抗原呈递，导致T细胞无法识别肿瘤细胞，以及产生免疫抑制蛋白[如PD-L1、转化生长因子-β（transforming growth factor-β, TGF-β）和白细胞介素-10（interleukin-10, IL-10）]来刺激肿瘤生长并阻断共刺激信号，抑制T细胞功能^[15,16]^。

PD-1/PD-L1抑制剂通过阻断PD-1/PD-L1免疫抑制通路，解除对于T细胞免疫功能的抑制^[17]^，但当肿瘤细胞MHC I类分子下调时，免疫逃逸发生，免疫耐药出现^[16]^。不同于PD-1/PD-L1抑制剂，TCE通过直接连接T细胞与肿瘤细胞，在T细胞和肿瘤细胞之间形成免疫突触，在不依赖于MHC I类分子呈递抗原的情况下，直接激活T细胞，引导T细胞定向释放穿孔素与颗粒酶B等细胞毒性效应分子，诱导肿瘤细胞发生溶解和凋亡，因此能够克服肿瘤MHC下调的问题^[18,19]^。同时，TCE还可促进T细胞增殖，增加肿瘤局部效应T细胞数量，从而进一步增强抗肿瘤效应^[20,21]^。

### 2.2 DLL3-TCE药物的作用机制和研发现状

Notch信号通路是一种高度保守的细胞间信号通路，参与包括肺神经内分泌细胞等多种细胞的发育过程^[22]^。DLL3是Notch受体的抑制性配体，通过阻断Notch信号传导，诱导NEC细胞异常生长^[23]^。在正常组织中，DLL3表达水平极低，且主要表达在细胞质内^[24]^，而在NEC，尤其是SCLC中，DLL3在细胞表面异常高表达，阳性率可达85%-96%^[25]^。这一特性使其成为SCLC极具潜力的治疗靶点。

靶向DLL3的TCE，其一端可特异性识别并结合SCLC肿瘤细胞表面高度表达的抗原DLL3，另一端同时结合T细胞表面的CD3受体^[9]^，进而衔接肿瘤细胞和T细胞，在两者之间形成免疫突触，激活T细胞杀伤肿瘤细胞。基于这种特殊的药物结构设计，DLL3-TCE同时呈现出了免疫治疗的长期生存、持久缓解与靶向治疗的高缓解、低毒性的双重优势。

以塔拉妥单抗为代表的DLL3-TCE药物，基于DeLLphi-301^[25]^、DeLLphi-304^[26]^、DeLLphi-307^[27]^研究取得的积极结果，已获得美国食品药品监督管理局（Food and Drug Administration, FDA）和中国国家药品监督管理局（National Medical Products Administration, NMPA）批准用于复发SCLC患者的治疗。同时基于其在后线治疗中取得的突破，针对一线ES-SCLC和局限期SCLC（limited-stage SCLC, LS-SCLC）的III期研究正在进行中，有望为SCLC治疗带来新的突破。除此之外，多款DLL3-TCE药物^[28-31]^，包括Obrixtamig（BI 764532）、Gocatamig（HPN328）、Alveltamig（ZG006）、SHR-7787、HLX-3901及RO7616789，在SCLC和NEC的探索正在进行中（表3）。

## 3 DLL3-TCE药物在SCLC中的临床应用推荐

### 3.1 DLL3-TCE药物在复发SCLC中的治疗推荐

**专家共识1：推荐塔拉妥单抗作为含铂化疗失败的ES-SCLC二线及后线治疗的优选方案。且无论亚裔/非亚裔，无论先前是否接受过ICIs治疗，铂敏感复发（无化疗间期≥90天）或铂耐药复发（无化疗间期<90天）患者，塔拉妥单抗均显示一致临床获益（证据水平：1类，推荐级别：强）。**

II期临床研究DeLLphi-301^[25]^和III期临床研究DeLLphi-304^[26]^一致证实塔拉妥单抗在复发SCLC患者中的临床获益和良好的安全性。DeLLphi-301研究^[25]^纳入既往接受过二线或以上治疗后的SCLC患者，10 mg剂量组纳入的100例患者中，经确认的客观缓解率（objective response rate, ORR）为40%，中位缓解持续时间（duration of response, DoR）为9.7个月，中位OS达到15.2个月（中位随访时间为16.6个月）。DeLLphi-301研究^[25]^突出的疗效获益支持塔拉妥单抗作为SCLC三线及以上治疗的优选方案。DeLLphi-304研究^[26]^纳入了既往接受过一线铂类化疗后进展的ES-SCLC患者，共计入组509例，中期分析结果（中位随访时间为11.2个月）显示塔拉妥单抗相较二线标准化疗方案（包括：拓扑替康、伊利替康、芦比替定等）显著改善患者的OS[中位OS：13.6 *vs* 8.3个月；风险比（hazard ratio, HR）=0.60，95%CI: 0.47-0.77，*P*<0.001]、ORR（35% *vs* 20%）及无进展生存期（progression-free survival, PFS）（中位PFS：4.2 *vs* 3.7个月，HR=0.71，*P*=0.002），并降低≥3级治疗相关AEs（treatment related AEs, TRAEs）发生率（27% *vs* 62%），同时显著改善了患者呼吸困难和咳嗽等症状。DeLLphi-304研究证据支持塔拉妥单抗作为SCLC二线治疗的优选方案。值得关注的是，由于复发SCLC患者通常疾病进展迅速，推后使用塔拉妥单抗可能导致错失治疗机会。因此，在条件允许的情况下，建议优先在二线治疗阶段使用塔拉妥单抗。

DeLLphi-301、DeLLphi-304、DeLLphi-307等研究及亚组分析^[25-27,32]^进一步探索了塔拉妥单抗在亚裔和非亚裔、前线ICIs初治和经治、铂敏感复发（无化疗间期≥90天）或铂耐药复发（无化疗间期<90天）亚组人群中的临床获益。DeLLphi-301和DeLLphi-304为全球多中心研究，均分析了亚裔患者获益情况。DeLLphi-301研究^[32]^共入组43例亚裔患者，经确认的ORR为46.3%，中位DoR为7.2个月，中位OS为19个月，与总人群获益相似。DeLLphi-304研究^[26]^共入组204例亚裔患者，亚裔患者OS亚组分析证实塔拉妥单抗对比化疗与总人群显示一致获益（HR=0.75, 95%CI: 0.50-1.11）。DeLLphi-307^[27]^是一项在中国开展的II期桥接研究，共纳入31例既往接受过二线或以上治疗的SCLC患者，研究达到主要终点ORR，经确认的ORR为39%，同样显示出良好的抗肿瘤活性。以上临床证据支持塔拉妥单抗在亚裔患者中良好的获益。DeLLphi-304研究^[26]^分析了一线PD-1/PD-L1抑制剂初治和经治患者的临床获益，该研究纳入患者中70.5%既往接受过PD-1/PD-L1抑制剂治疗，亚组分析结果显示，相比化疗，塔拉妥单抗在一线未接受PD-1/PD-L1抑制剂治疗的患者（mOS：13.6 *vs* 8.3个月；HR=0.65，95%CI: 0.42-1.03）和一线接受了PD-1/PD-L1抑制剂治疗的患者（mOS：14.1 vs 8.3个月；HR=0.61，95%CI: 0.45-0.82）中均显示出良好的OS改善，提示无论先前是否接受过ICIs治疗，塔拉妥单抗用于复发SCLC均一致获益。ES-SCLC根据铂类化疗后距疾病复发的时间间隔分为铂敏感复发（无化疗间期≥90天）和铂耐药复发（无化疗间期<90天），铂耐药复发患者预后更差^[33,34]^。DeLLphi-301研究^[25]^分析了铂耐药复发和铂敏感复发患者的生存情况，研究发现塔拉妥单抗均带来相似的OS获益。无化疗间期（<90 *vs* ≥90至<180 *vs* ≥180天）是DeLLphi-304研究^[26]^中的分层因素，亚组分析显示，与化疗组相比，塔拉妥单抗在铂耐药复发患者（mOS：10.9 *vs* 6.4个月；HR=0.60，95%CI: 0.43-0.84）和铂敏感复发患者（mOS：17.1 *vs* 10.6个月；HR=0.65，95%CI: 0.45-0.93）中均显示更佳的OS获益。提示无论铂敏感复发还是铂耐药复发患者，塔拉妥单抗治疗疗效均优于标准化疗。

除塔拉妥单抗外，其他DLL3-TCE在复发SCLC患者中也正在开展早期探索。Obrixtamig（BI 764532）在I期研究^[29]^中纳入168例经治SCLC和其他NEC患者，其中SCLC占比为49.4%，Obrixtamig单药治疗后，SCLC患者经确认的ORR为20%。DAREONTM-9研究^[35]^是一项评估Obrixtamig联合拓扑替康治疗经治SCLC的Ib期临床研究，共纳入25例患者，经确认的ORR为69%，中位DoR尚未达到。Gocatamig（MK-6070/HPN328）在一项I/II期临床研究^[30]^中纳入了73例经治ES-SCLC和其他NEC患者，其中在Gocatamig 24 mg剂量组中，25例SCLC患者经确认的ORR为46%。Alveltamig在一项II期临床研究^[31]^探索了用于既往接受过至少二线治疗的SCLC患者，在中位随访时间为9个月时，10 mg剂量组中30例患者经确认的ORR为53.3%，中位PFS为7个月，中位DoR和OS尚未达到；Alveltamig对比拓扑替康用于复发SCLC的III期临床研究（CTR20253783, NCT07189455）也正在进行中。以上研究提示其他DLL3-TCE用于复发ES-SCLC也观察到了良好的早期信号，期待更大样本量和更长时间的随访以进一步明确生存获益。

### 3.2 DLL3-TCE药物在伴脑转移的复发SCLC的治疗推荐

**专家共识2：推荐塔拉妥单抗作为伴无症状或经治稳定脑转移的复发SCLC患者的标准治疗选择之一，塔拉妥单抗已有证据初步显示出良好的颅内抗肿瘤活性，且较化疗能延长脑转移患者的生存（证据水平：2类，推荐级别：强）。**

脑转移是SCLC患者最常见的远处转移部位。SCLC患者初诊时脑转移发生率约为10%；在疾病治疗和进展过程中，脑转移发生率可高达40%-50%^[36]^。既往研究^[37]^表明，脑转移SCLC患者总体预后不佳，中位OS仅约6个月，亟待更有效的治疗手段。DeLLphi-301研究^[38]^纳入了23例经治稳定的脑转移患者，这部分患者接受塔拉妥单抗治疗ORR达到52%，中位OS达到14.3个月，其中17例患者基线脑转移病灶≥10 mm，经塔拉妥单抗治疗后根据改良版神经肿瘤脑转移缓解评估（modified response assessment in neuro-oncology brain metastases, mRANO-BM）的颅内ORR达到30%，颅内疾病控制率（disease control rate, DCR）达到94%，塔拉妥单抗显示了良好的颅内抗肿瘤活性。DeLLphi-304研究^[26]^则允许同时纳入伴无症状或经治稳定脑转移的患者，研究共入组228例（塔拉妥单抗组113例，化疗组115例）脑转移患者，是否存在脑转移也是研究的分层因素之一，脑转移亚组分析显示，塔拉妥单抗相比化疗可为脑转移患者带来更多OS获益（HR=0.45, 95%CI: 0.31-0.65），证实无症状或经治稳定脑转移患者均可从塔拉妥单抗治疗中获得生存提升。

塔拉妥单抗用于有症状脑转移患者的研究证据有限，一项塔拉妥单抗的真实世界研究病例报道^[39]^共纳入10例有症状但未经脑放疗的脑转移患者（包括1例怀疑软脑膜转移），其中9例（90%）在塔拉妥单抗治疗后的第一次脑磁共振成像（magnetic resonance imaging, MRI）检查中显示颅内病灶缩小或稳定、快速且显著的影像学缓解和临床症状改善，提示可减少或推迟对全脑放疗的需求。

目前塔拉妥单抗用于SCLC脑转移治疗的作用机制尚不完全明确，研究^[40]^提示，脑转移病灶引发的局部炎症反应可能破坏血脑屏障的完整性，并上调黏附分子的表达，从而促进活化的T细胞向脑部病灶浸润，因此在既往有关ICIs的研究中亦观察到一定的颅内活性，塔拉妥单抗的颅内抗肿瘤作用机制仍有待进一步研究明确。此外，对于合并脑转移的SCLC患者，放疗是重要的治疗手段，塔拉托单抗与放疗的应用时序问题尚缺乏充足的临床证据支持，需要更多前瞻性研究加以验证与回答。

### 3.3 DLL3-TCE药物在一线ES-SCLC和LS-SCLC治疗中的前景

**专家共识3：ES-SCLC一线化免诱导治疗阶段开始联合DLL3-TCE药物或免疫维持治疗阶段开始联合DLL3-TCE药物的早期探索均显示突出的临床获益，III期确证性研究正在进行中；LS-SCLC同步放化疗后DLL3-TCE药物巩固治疗进入III期研究阶段，推荐符合条件的SCLC患者加入相关临床研究（证据水平：2类，推荐级别：强）。**

ICIs的出现显著改善了SCLC患者的生存获益，PD-1/PD-L1抑制剂联合含铂化疗已成为ES-SCLC一线标准治疗方案^[3]^，但疗效仍有待进一步提升^[2,4,5]^，在一线化免治疗的诱导阶段或维持阶段联合DLL3-TCE是临床中探索的热点方向。DLL3-TCE作为一种能够直接募集并激活T细胞的新型药物，在作用机制上与PD-1/PD-L1抑制剂具有潜在协同作用：前者可直接促进T细胞的活化，后者则可解除对T细胞活性的抑制^[41]^。临床前研究^[42]^表明，T细胞在通过TCE介导杀伤肿瘤过程中，T细胞表面PD-1和肿瘤细胞表面PD-L1表达会上调，联合PD-L1抑制剂可进一步增强T细胞的抗肿瘤活性。

DeLLphi-303为一项多中心、多队列Ib期临床研究，探索了塔拉妥单抗联合PD-L1抑制剂作为一线维持治疗或一线诱导和维持治疗用于ES-SCLC的可行性。维持治疗队列纳入88例一线化免诱导治疗后未进展的ES-SCLC患者^[43]^，入组后接受塔拉妥单抗联合阿替利珠单抗（*n*=48）或度伐利尤单抗（*n*=40）作为维持治疗，中位随访18.4个月，中位OS达到25.3个月，1年OS率为82%，展示出生存提升的巨大潜力。诱导+维持治疗队列纳入96例患者^[44]^，接受塔拉妥单抗联合化疗及阿替利珠单抗（*n*=56）或度伐利尤单抗（*n*=40）诱导治疗，随后接受塔拉妥单抗联合阿替利珠单抗或度伐利尤单抗维持治疗，中位随访13.8个月，ORR达到71%，中位DoR为11个月；中位OS尚未达到，1年OS率为80.6%，也展示出良好的疗效潜力，同时两个队列均显示良好的安全性。目前塔拉妥单抗联合度伐利尤单抗用于ES-SCLC一线维持治疗的国际多中心III期临床研究DeLLphi-305（NCT06211036）已完成入组，正在随访中；塔拉妥单抗联合度伐利尤单抗和含铂化疗用于ES-SCLC一线诱导和维持治疗的国际多中心III期临床研究DeLLphi-312（NCT07005128）正在进行中，期待两项III期临床研究进一步证实塔拉妥单抗联合PD-L1抑制剂用于ES-SCLC一线治疗的疗效及安全性。

多个在研的DLL3-TCE药物也在进行一线联合治疗的探索。DAREON-8是一项I期剂量递增/扩展研究^[45]^，评估Obrixtamig联合阿替利珠单抗用于ES-SCLC患者一线诱导和维持治疗的疗效及安全性，中位随访6.9个月，结果显示，经确认的ORR为68%，中位DoR为7.3个月，中位PFS尚未达到，9个月PFS率为52%，安全性良好。Obrixtamig联合阿替利珠单抗和含铂化疗用于ES-SCLC一线诱导和维持治疗的III期确证性研究（NCT07472517）正在进行中。此外，Alveltamig联合斯鲁利单抗用于一线ES-SCLC治疗的Ib期研究（CTR20254357）、Gocatamig联合B7-H3抗体偶联药物（antibody-drug conjugate, ADC）用于一线ES-SCLC免疫和化疗诱导治疗后维持治疗的I期研究正在进行中（NCT07231445）。

除布局ES-SCLC外，DLL3-TCE在LS-SCLC领域的探索也正在进行中。同步放化疗既往是LS-SCLC的标准治疗方式，然而大部分患者会在治疗的2年内出现复发，5年生存率仅为29%-34%^[46]^。在过去的30年中，LS-SCLC的系统治疗未取得任何进展，直至ADRIATIC研究的成功证实同步放化疗后进行免疫巩固治疗可进一步延长LS-SCLC患者的生存^[47,48]^。DeLLphi-306（NCT06117774）是一项全球多中心III期临床研究，旨在探索同步放化疗后塔拉妥单抗单药巩固治疗对比安慰剂治疗LS-SCLC的疗效及安全性，研究正在进行中。相信未来DeLLphi-306研究将为同步放化疗后的LS-SCLC患者提供更多治疗选择。

### 3.4 DLL3-TCE药物是否需要进行DLL3筛选

**专家共识4：基于DLL3在SCLC中高表达，结合目前研究证据且临床研究中未将DLL3表达作为入组和用药标准，故目前应用DLL3-TCE药物治疗SCLC时，无需进行DLL3检测；DLL3表达的预测价值仍待更多研究探索（证据水平：1类，推荐级别：强）。**

DLL3在正常组织中表达水平极低，且主要位于细胞质内^[24]^；而在SCLC中，DLL3可异常表达于SCLC细胞表面^[24,49]^。目前，来自塔拉妥单抗的多项研究^[25,26,43,50]^的回顾性分析显示，高达93%-97%的基线SCLC肿瘤组织中DLL3蛋白表达呈阳性。在一项有关Obrixtamig的I期研究^[29]^中，94%复发SCLC患者筛选期DLL3检测呈阳性。此外，有关Alveltamig的ZG006-002研究^[31]^共纳入了60例中国SCLC患者，DLL3的阳性率为95%，再次证实了DLL3在SCLC中的高表达。

有关塔拉妥单抗临床研究回顾性分析了不同DLL3表达水平患者的肿瘤缓解情况。DeLLphi-301研究^[25]^中观察到，在DLL3表达阳性、阴性以及未进行DLL3检测的患者中，塔拉妥单抗治疗均显示良好的肿瘤缓解，目前其说明书中未要求用药前进行DLL3表达的检测^[51]^。此外，临床前研究^[13]^显示塔拉妥单抗能够有效激活T细胞，即使在SCLC细胞表面DLL3表达水平极低的情况下（<1000 molecules/cell），也显示出对SCLC细胞的显著杀伤作用，提示DLL3表达水平可能并非指导疗效获益的决定性因素。故目前研发中的多个DLL3-TCE（Obrixtamig、Alveltamig、Gocatamig等）药物，临床研究设计中均未限制入组患者的DLL3表达水平^[29-31]^。

目前，DLL3尚无标准化的检测方法，仍处于科研探索阶段，临床研究^[25-27,29-31]^主要应用了基于组织标本的免疫组织化学（immunohistochemistry, IHC）方法进行检测，基于血液标本的循环肿瘤细胞检测等方法也正在探索中，标准化的检测方法与明确的阳性判断标准有待未来进一步完善。因此，鼓励开展科研探索，挖掘可用于预测DLL3-TCE药物疗效的生物标志物，在条件允许的情况下可考虑检测DLL3表达水平，以深入探索DLL3与DLL3-TCE药物临床治疗获益的相关性，同时也鼓励扩展探索其他有效的预测性标志物。

## 4 DLL3-TCE药物的AEs特征

现有的临床证据^[25,26,51]^显示，TCE药物用于SCLC患者的安全性总体可控，常见的治疗期间出现的AEs（treatment emergent AEs, TEAEs）包括CRS、食欲减退、发热、味觉障碍、便秘、疲劳、恶心和血液学毒性等（表4）。其中，味觉障碍为DLL3-TCE药物较为特殊的AEs，但均为低级别；CRS和ICANS是TCE药物需要特殊关注的，区别于传统化疗、靶向治疗和ICIs治疗的AEs。神经内分泌转录因子ASCL1驱动某些类型味蕾细胞的晚期分化^[52]^，由于ASCL1还调控DLL3的表达^[53]^，因此味蕾细胞也可能表达DLL3。DLL3-TCE可能会导致T细胞介导的对表达DLL3的味蕾细胞的破坏，从而引发味觉障碍^[54]^。在塔拉妥单抗DeLLphi-301研究^[26]^中，味觉障碍的发生率为32%，中位发生时间为34天；在DeLLphi-304研究^[27]^中味觉障碍的发生率为23%，均为1-2级，中位发生时间为28天，无塔拉妥单抗导致治疗终止的情况发生。其他DLL3-TCE药物Obrixtamig和Gocatamig的早期临床研究^[29,30]^中显示味觉障碍的发生率为23%-45%，级别同样也都为1-2级。

**表4:** 基于DeLLphi-300（*n*=88）、DeLLphi-301（*n*=133）和DeLLphi-304（*n*=252）研究473例接受塔拉妥单抗治疗患者的安全性分析

最常发生的TEAEs（≥15%）	总体TEAEs发生率（*n*=473），*n*（%）	≥3级TEAEs发生率（*n*=473），*n*（%）
CRS	268 (56.7)	9 (1.9)
食欲减退	172 (36.4)	9 (1.9)
发热	151 (31.9)	3 (0.6)
味觉障碍	148 (31.3)	0 (0.0)
便秘	144 (30.4)	2 (0.4)
贫血	142 (30.0)	22 (4.7)
疲劳	141 (29.8)	20 (4.2)
恶心	118 (24.9)	4 (0.8)
虚弱	90 (19.0)	14 (3.0)
中性粒细胞减少症	80 (16.9)	39 (8.2)
低钠血症	79 (16.7)	28 (5.9)
淋巴细胞减少症	74 (15.6)	50 (10.6)
头痛	77 (16.3)	0 (0.0)
呼吸困难	52 (11.0)	10 (2.1)

TEAEs：治疗期间出现的不良事件。

CRS是一种全身性炎症反应，其发生主要源于T细胞在识别并攻击癌细胞时，会释放效应性细胞因子[如干扰素-γ（interferon-γ, IFN-γ）、肿瘤坏死因子-α（tumor necrosis factor-α, TNF-α）等]，继而诱导促炎性细胞因子（如IL-6、IFN-γ、IL-10等）持续释放，导致免疫系统过度激活，从而引发系统性炎症反应^[54]^。CRS的典型症状为发热，随着症状进行性发展，可能出现低血压、缺氧，也可能伴随其他全身症状（包括但不限于呼吸急促、心动过速、头痛、恶心、呕吐及终末器官功能异常）^[55]^。目前，CRS的分级主要参考美国移植与细胞治疗学会（American Society for Transplantation and Cellular Therapy, ASTCT）分级标准^[55]^，按照发热、低血压、缺氧的情况和严重程度进行划分，1-4级分级标准详见表5^[51,54,55]^，5级为因CRS导致的死亡。TCE药物相关的CRS主要为1-2级，多发生于首次或第2次给药后^[54]^。DeLLphi-300、DeLLphi-301和DeLLphi-304研究汇总分析了473例接受塔拉妥单抗治疗患者的安全性数据^[51]^，其中任意级别CRS发生率为57%，≥3级CRS发生率仅为1.9%，中位发生时间为16 h[四分位距（interquartile range, IQR）：9-26.5 h]，主要发生在用药早期阶段。其他DLL3-TCE药物早期研究中也观察到相似的CRS发生率和发生时间。Obrixtamig针对168例二线及以后SCLC及其他NEC的I期研究^[29]^中，任意级别CRS发生率为57%，≥3级CRS发生率为3%，中位发生时间为16 h（范围：3-54 h）。Alveltamig和Gocatamig^[30,31]^在复发SCLC的I/II期研究观察到相似的趋势，任意级别CRS发生率分别为40%和55%，≥3级发生率分别为3%和1%。

**表5:** CRS分级标准和管理策略

分级*	严重程度描述	管理策略
1级	发热≥38 ^o^C，无低血压或缺氧	针对发热进行对症治疗（如对乙酰氨基酚） 考虑地塞米松4-10 mg（或等效药物）口服或静脉给药
2级	发热: ≥38 ^o^C 低血压: 补液治疗有效，不需要血管升压药 低氧血症: 仅需低流量吸氧（≤6 L/min）	建议住院治疗并监测发热、低血压和缺氧，根据需要使用脉搏血氧测定或心电监测，至少24 h 针对发热进行对症治疗（如对乙酰氨基酚） 考虑地塞米松8 mg（或等效药物）^#^口服或静脉给药 根据指征吸氧和静脉输液[室内空气下血氧饱和度<90%时，低流量（≤6 L/min）吸氧； 收缩压<85 mmHg时静脉补液。持续性心动过速（如>120 bpm）也可能提示需要对低血压进行干预] 考虑使用托珠单抗（或等效药物） 重新开始下一个计划剂量治疗时，从开始输注起，患者在适当医疗机构接受22-24 h监测
3级	发热: 体温≥38 ^o^C 低血压: 需一种血管升压药±血管加压素 低氧血症: 需高流量吸氧（>6 L/min）， 面罩吸氧	除针对2级CRS的治疗外 建议加强监护，如ICU监护 地塞米松8 mg（或等效药物）^#^静脉给药，每8 h一次，最多给药3次 必要时给予血管加压药支持治疗（血管升压药±血管升压素） 根据需要进行高流量吸氧治疗或面罩吸氧 建议使用托珠单抗（或等效药物）（托珠单抗：单次给药剂量为4-8 mg/kg， 每次输注最大剂量为800 mg，托珠单抗可再重复给药3次，两次给药间隔至少8 h） 重新开始下一个计划剂量治疗时，从开始输注起，患者在适当医疗机构接受22-24 h监测
4级	发热: 体温≥38 ^o^C 低血压: 需血管加压素以外的多种血管升压药 低氧血症: 需机械辅助通气	进行针对3级CRS的治疗 ICU监护 必要时可给予多种血管升压药支持治疗 根据需要供氧 建议使用托珠单抗（或等效药物）（托珠单抗：单次给药剂量为4-8 mg/kg， 每次输注最大剂量为800 mg，托珠单抗可再重复给药3次，两次给药间隔至少8 h）

*基于美国移植与细胞治疗学会（ASTCT）共识分级（2019版）；^#^按照标准治疗指南逐渐减低类固醇用量。 ICU：重症监护室。

ICANS是TCE治疗中另一种需要关注的特殊AE，但其病理生理机制尚不清楚，可能与多种因素共同作用有关，包括炎性细胞因子增加血管通透性、内皮细胞激活导致血脑屏障破坏、脑脊液中细胞因子水平升高^[56,57]^。ICANS可能与CRS同时发生，也可能在CRS之后发生。ICANS的临床表现多变，可能包括但不限于意识水平下降、失语、认知功能受损等^[57]^。目前，ICANS的分级主要采用ASTCT分级标准^[55]^，综合免疫效应细胞相关脑病（immune effector cell-associated encephalopathy, ICE）评分（表6）、意识水平、癫痫发作、运动障碍以及颅内压升高/脑水肿事件发生情况进行划分，1-4级的分级标准详见表7（根据最严重的事件确定分级）^[51,54,55]^，5级为因ICANS导致的死亡。TCE药物相关的ICANS发生率较低，大多为1-2级，多发生在用药前2个周期^[54]^。在塔拉妥单抗DeLLphi-300、DeLLphi-301和DeLLphi-304研究汇总分析中，473例接受塔拉妥单抗治疗患者的任意级别ICANS发生率为4.7%，≥3级ICANS发生率仅为0.2%^[51]^，ICANS的中位发生时间为9天（IQR：2-13天），主要发生在用药的前2个周期^[26,27]^。其他DLL3-TCE药物（Obrixtamig、ZG006和Gocatamig）的早期临床研究中，也观察到相似的趋势，ICANS整体发生率较低，且以1-2级为主（任意级别ICANS发生率：1.7%-9%，≥3级ICANS发生率：0%-3%）^[29-31]^。

**表6:** ICE评估

ICE评估工具	得分
定向力：年份、月份、城市、医院	4
命名能力：说出3种物体名称的能力（例如指向时钟、笔、按钮）	3
执行命令能力：执行简单指令的能力（例如："伸出两根手指"或"闭上眼睛并伸出舌头"）	1
书写能力：书写标准句子的能力（例如："我们的国旗是五星红旗"）	1
注意力：从100每隔10个数倒数	1

ICE：免疫效应细胞相关脑病。

**表7:** ICANS分级标准和管理策略

分级*	症状或体征	管理策略
1级	ICE评分7-9分 意识水平无下降	支持治疗
2级	ICE评分3-6分 和/或轻度嗜睡，易被声音唤醒	支持治疗 地塞米松8-10 mg（或等效药物）^#^口服或静脉给药；若症状加重，可每12 h重复给药，或甲泼尼龙1 mg/kg（或等效药物）^#^静脉给药，每12 h一次 监测神经系统症状，并考虑咨询神经科医生和其他专家进行进一步评价和管理 在下一次给药后监测患者22-24 h
3级	ICE评分0-2分 和/或意识水平下降，仅可被触觉刺激唤醒 和/或任何可迅速消退的局灶性或全身性临床惊厥发作 或在干预后消退的非痉挛性惊厥发作 (EEG提示) 和/或神经影像学显示局灶性或局部水肿	建议加强监护，如ICU监护 考虑进行机械通气以保护气道 地塞米松10 mg（或等效药物）^#^静脉给药，每6 h给药一次，或甲泼尼龙1 mg/kg（或等效药物）^#^静脉给药，每12 h给药一次 如果患者持续出现≥3级神经毒性，则考虑每2-3天重复神经影像学检查（CT或MRI）在下一次给药后监测患者22-24 h
4级	ICE评分0分（患者无法唤醒，无法进行ICE评估） 和/或木僵或昏迷 和/或危及生命的持续性癫痫（>5 min） 重复性临床或电刺激惊厥发作，但两次发作之间未恢复到基线水平 和/或神经影像学显示弥漫性脑水肿, 去脑状态或去皮质体位或视神经乳头水肿、颅神经VI麻痹，或库欣三联症	ICU监护 考虑进行机械通气以保护气道 高剂量皮质类固醇（如甲泼尼龙^#^1000 mg/d，分次静脉给药，持续3天） 若患者持续出现≥3级神经毒性，则考虑每2-3天重复神经影像学检查（CT或MRI）根据机构指南治疗癫痫抽搐状态

*基于美国移植与细胞治疗学会（ASTCT）共识分级（2019版）；^#^按照标准治疗指南逐渐减低类固醇剂量。 EEG：脑电图；CT：计算机断层扫描；MRI：磁共振成像。

总体来说，CRS和ICANS的发生具有可预测、可管理的特点。首先，不同于ICIs治疗的免疫相关AEs（immune-related AEs, irAEs）累及全身多器官、临床表现与发生时间多样，CRS和ICANS的发生具有高度特异性，多发生于治疗早期^[58]^。其次，TCE药物引起的CRS和ICANS多为1-2级，与嵌合抗原受体T细胞（chimeric antigen receptor T-cell, CAR-T）疗法相比，≥3级CRS（1%-3% vs 8.8%）和ICANS发生率（0%-3% vs 12.5%）明显更低^[59]^，通过早期监测、早期识别、及时干预和规范管理，更为可控且可管理，因CRS和ICANS导致治疗终止的比例低（仅约3%）。需要注意的是，目前的安全性特征主要基于临床研究数据，同时尚需进一步关注真实世界中DLL3-TCE药物的安全性表现，及时进行干预处理，确保患者用药安全。

## 5 DLL3-TCE药物的早期用药与监测

### 5.1 DLL3-TCE药物AEs的早期预防

**专家共识5：基于DLL3-TCE药物相关的CRS和ICANS多发生于治疗初期，在第1周期通过分步剂量给药、皮质类固醇预先用药等措施可以早期降低CRS和ICANS的发生风险（证据水平：1类，推荐级别：强）。**

基于CRS和ICANS多发生于治疗初期的特征^[54]^，早期预防和监测对于尽早识别并及时干预，避免CRS和ICANS恶化至关重要。结合既往血液肿瘤TCE药物和CAR-T疗法的研发与临床应用经验，目前临床常用的早期预防策略主要包括：分步剂量给药、预防性皮质类固醇用药及补液等支持治疗^[60,61]^。

既往血液肿瘤TCE药物研究发现，分步剂量的给药策略能够降低CRS和ICANS的发生率和严重程度。早期Blinatumomab研究^[60]^显示，与固定剂量方案相比，分步剂量给药可显著降低细胞因子分泌水平。随后Tebentafusp研究^[61]^发现，采用分步剂量给药方案时，其推荐的II期剂量相较采用固定每周剂量方案的最大耐受剂量提高36%。进一步的Mosunetuzumab药效学分析^[62]^亦表明，炎性细胞因子的释放主要与首次给药有关，而在后续更高剂量给药过程中IL-6水平并未进一步升高。上述证据支持在首次给药时采用较低起始剂量，并逐步递增至目标剂量，以“渐进式”启动免疫反应，避免药物浓度快速达到峰值进而诱发免疫过度激活，从而降低CRS和ICANS风险及其严重程度。

既往CAR-T疗法研究^[63]^发现，皮质类固醇激素能够降低CRS和ICANS的发生率和严重程度。ZUMA-1研究^[64]^评估了皮质类固醇激素的预防性使用对接受阿基仑赛治疗的复发/难治性大B细胞淋巴瘤患者发生CRS和神经系统AEs的影响。结果显示，与未给予预防性皮质类固醇激素治疗相比，预防性使用皮质类固醇激素不仅可降低≥3级CRS（0% *vs* 13%）和神经系统AEs（13% *vs* 28%）的发生率，也可降低毒性管理所需的激素累积剂量（1878 *vs* 7418 mg），且未影响疗效（ORR: 95% vs 83%）。目前TCE药物多采用第1周期预防性使用皮质类固醇来降低CRS和ICANS的发生率和严重程度。此外，静脉补液有助于扩充血容量，降低低血压风险，防止CRS恶化；部分药物通过给予抗组胺药和解热剂（如对乙酰氨基酚）预处理来预防CRS相关症状发生或恶化^[65]^。

目前，塔拉妥单抗说明书^[51]^推荐第1周期采用分步剂量给药方案（第1天1 mg，第8和15天10 mg），并在第1和8天给药前1 h内静脉给予8 mg地塞米松（或等效药物）；输注完成后静脉输注1 L生理盐水（2-4 h），以降低CRS和ICANS的发生率和严重程度（图1）。其他在研DLL3-TCE药物的临床研究中同样采用了分步剂量和预防性用药的策略^[29-31]^，其中，Alveltamig在第1周期给药前的预防性用药除皮质类固醇激素外^[31]^，还同时联合应用了抗组胺药及解热镇痛药（如对乙酰氨基酚），以降低CRS的发生风险。

**图1:**
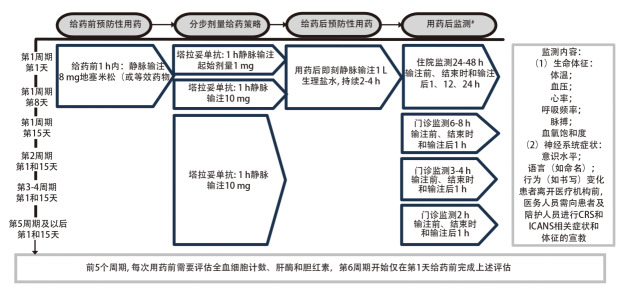
塔拉妥单抗的给药和监测策略 每28天为1个周期。^#^在第1周期第1和8天，从开始输注起的48 h期间，如患者离开医院，则建议患者的附近有可及的适当的医疗机构（1 h路程），且有陪护人员陪同；自第1周期第1和8天之后，如患者既往治疗中未出现≥2级CRS、ICANS或其他神经系统毒性，则无需延长在医疗机构的监测时间。

### 5.2 DLL3-TCE药物输注的监测推荐及出院后的患者教育

**专家共识6：早期监测能够及时发现CRS和ICANS，生命体征的监测能够及时发现CRS，患者意识、行为、语言的监测能够及时发现ICANS；推荐早期（第1周期第1和8天，即C1D1和C1D8）住院治疗监测（24-48 h），稳定的患者后续可以选择门诊治疗和监测（2-6 h）；患者离开医院前需进行患者及陪护人员的居家监测宣教，同时建议前2个周期出院期间对患者进行电话随访，保证能及时发现迟发性CRS或ICANS，及时救治（证据水平：2类，推荐级别：强）。**

CRS和ICANS多发生在治疗早期^[54]^，给药后早期监测（C1D1和C1D8）有助于及早发现CRS和ICANS并及时干预。CRS通常以发热为首要表现，进行性出现低血压、缺氧等症状^[55]^，故生命体征的监测可帮助及早发现CRS的发生。ICANS的典型特征为意识水平下降^[55]^，对神经系统相关表现的动态评估[包括患者意识、行为（如书写）、语言（如命名）等变化]有助于早期识别ICANS。

塔拉妥单抗在多项临床研究中探索了不同的输注后监测方案，在保证患者安全性的前提下探索缩短监测时间和减少住院的可行性。主要包括3种模式：（1）48 h住院监测：在DeLLphi-304研究^[26]^中，C1D1和C1D8输注后患者在适当医疗机构接受48 h监测。住院期间，输注后4 h内每30 min、4-8 h每1 h，以及24 h及24 h后，进行生命体征和神经系统症状的监测。研究结果支持48 h住院监测能保证良好的安全性。（2）24 h住院监测+患者指导：在DeLLphi-307研究^[27]^中，C1D1和C1D8输注后患者在适当医疗机构接受22-24 h监测。输注结束时、输注后1、12和24 h分别进行生命体征和神经系统症状的监测。同时出院前要求对患者及陪护人员进行CRS和ICANS宣教，用药后48 h内停留在距医院车程1 h范围内。研究结果支持24 h住院监测能保证良好的安全性。（3）门诊监测+患者指导：DeLLphi-300研究^[66]^中探索了48 h住院监测对比6-8 h门诊监测（用药后48 h内停留在距医院车程1 h范围内）的安全性，结果显示两组总体TRAEs发生率及CRS和ICANS发生特征均相似，初步支持门诊监测的可行性。正在进行的III期研究DeLLphi-305（NCT06211036）、DeLLphi-306（NCT06117774）和DeLLphi-312（NCT07005128）采用了门诊监测方案，C1D1和C1D8输注后观察1-2 h，若无CRS、ICANS和其他急性症状则可考虑出院。出院前要求对患者及陪护人员进行CRS和ICANS宣教并发放随身提示卡。24 h内停留在距医院车程1 h范围内，陪护人员24 h看护，12 h体温监测，期待这些研究监测方案及安全性的详细数据公布。

综上，鉴于DLL3-TCE药物首次在实体瘤中获批，在临床应用的初期阶段，建议第1周期给药后住院监测24-48 h，后续周期可根据情况缩短住院监测时间。疾病稳定的患者在后续周期可以选择门诊治疗和监测（2-6 h）。同时应重视对患者和陪护人员的居家监测宣教，以指导及时发现迟发性CRS或ICANS，及时就医。

## 6 DLL3-TCE药物的AEs管理

DLL3-TCE药物常见的AEs包括CRS、食欲减退、发热、味觉障碍、便秘、疲劳、恶心、血液学毒性和肝酶异常等^[25-27,51,54]^。其中消化道AEs、血液系统毒性和肝酶异常也常见于其他抗肿瘤治疗中，考虑使用对症治疗和/或根据当地临床惯例及时处理。同时用药期间也需关注患者是否出现超敏反应体征和症状，根据临床指征进行管理。味觉障碍为DLL3-TCE药物较为特殊的AEs^[54]^，但均为低级别，管理建议包括：保持良好的口腔卫生，维持充足的水分摄入（每天约1.5-2.0 L），定期咀嚼小豆蔻；每天至少2次嗅闻丁香和柠檬的气味（每次约15 s），以及营养师提供营养咨询服务^[54]^。此外，需要特别关注的CRS和ICANS，早期识别、准确评估和针对性处理能够有效避免AEs恶化，促进AEs恢复。必要时建立AEs管理的多学科诊疗（multidisciplinary treatment, MDT）机制，由肿瘤内科/呼吸科主诊医生牵头，根据CRS和ICANS发生特征，纳入重症监护室（intensive care unit, ICU）、影像科、神经内科等专科医生共同决策。

### 6.1 CRS的识别、评估及处理

**专家共识7：（1）CRS多发生在用药后的早期（C1D1和C1D8），根据发热出现时间、患者症状改变（血压下降、血氧饱和度下降、感染症状等）能够帮助鉴别诊断，当仍不能确定为CRS时，其他相关检查（如血液学检查、胸部影像学检查、微生物培养）可考虑作为辅助手段（证据水平：3类，推荐级别：强）。（2）怀疑CRS时建议及时进行评估、分级、针对性处理，并监测患者生命体征（血压、血氧饱和度、体温、心率和呼吸频率），1-2级CRS能够通过及时的对症支持治疗（退热、吸氧、补液等）缓解，如支持治疗症状控制不佳，在排除感染或抗感染治疗的基础上，建议尽早应用糖皮质激素和/或托珠单抗，可降低CRS恶化的风险（证据水平：1类，推荐级别：强）。**

CRS的典型临床表现为发热（体温≥38 ^o^C），但由于该表现缺乏特异性，临床应重视与其他原因所致发热（尤其是感染）的鉴别^[58,67]^。CRS与感染的鉴别可结合发生时间、临床症状及辅助检查综合判断^[67]^：与感染相关发热相比，CRS往往发生于治疗早期（C1D1和C1D8），通常在给药后4-16 h内出现。除发热外，CRS还可能呈进行性发展，出现低血压和缺氧等表现（动脉血氧饱和度<90%）。若仍难以判定，可考虑进行血常规、微生物培养及胸部影像学检查作为辅助手段，以帮助区分CRS与感染（图2）。

**图2:**
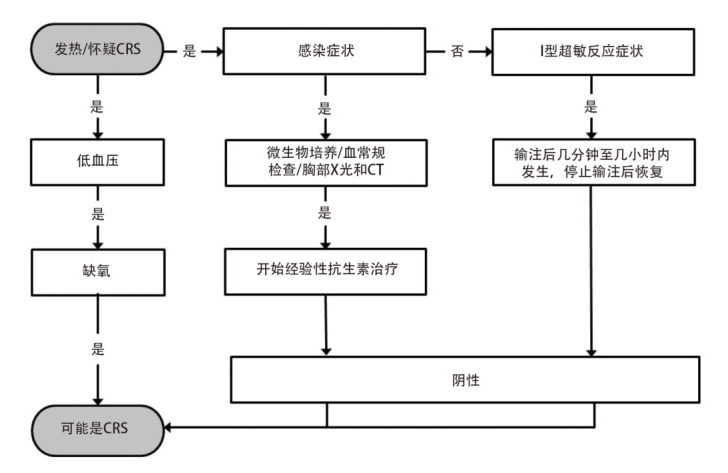
CRS和其他症状的鉴别诊断

对于高度怀疑或确诊为CRS的患者，应按照ASTCT标准进行分级^[55]^，并根据分级进行针对性处理（表5），并密切监测患者的生命体征（体温、血压、血氧饱和度、心率和呼吸频率），同时考虑对症支持治疗，包括退热和吸氧，也可考虑适时应用糖皮质激素和（或）托珠单抗。如果患者在输注4 h内出现相关症状，应立即启动3级CRS管理策略。

现有的临床研究证据显示，TCE引起的CRS主要为1-2级，绝大部分患者可通过对症支持治疗得到有效控制（如对乙酰氨基酚、静脉补液和糖皮质激素），仅少数患者需要额外干预措施（如托珠单抗），因CRS导致治疗终止的患者比例较低（附表1，http://www.lungca.org/files/2026s61s1.pdf）^[29-31,51]^，提示通过早期识别和及时干预，CRS可获得较好的转归。

### 6.2 ICANS的识别、评估及处理

**专家共识8：（1）ICANS多发生在用药的早期阶段（用药的前2个周期），通过发生时间、头颅MRI/CT等检查、神经科会诊，可帮助排除脑血管器质性改变或脑转移。大多数情况下，ICANS在暂停用药并给予糖皮质激素治疗后可得到恢复（证据水平：3类，推荐级别：强）。（2）如患者发生意识水平改变、语言/行为异常怀疑ICANS时，建议及时进行ICE评估、分级、针对性处理，尽早启动对症支持治疗和糖皮质激素治疗，早期识别和管理能够防止症状的加重，降低ICANS恶化的风险（证据水平：1类，推荐级别：强）。**

ICANS多发生于治疗的前2个周期，意识状态改变是其早期典型表现，若患者出现意识模糊、行为异常、语言障碍、书写能力减退等临床表现^[55]^，需高度警惕ICANS，建议及时进行ICE评估（表6）。按照ASTCT标准进行分级^[55]^，并根据分级进行针对性处理（表7）。ICANS患者的临床表现易与其他神经系统疾病（如脑血管器质性病变、肿瘤脑转移等）相混淆，一旦患者出现神经毒性相关临床表现，可结合发生时间、脑影像学检查，必要时神经科会诊，排除脑血管疾病或脑转移^[67,68]^（图3）。

**图3:**
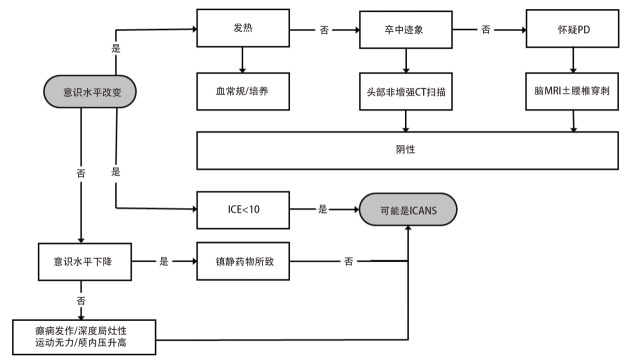
ICANS的鉴别诊断 PD：疾病进展。

对于高度怀疑ICANS的患者，建议尽早启动支持治疗和糖皮质激素治疗。现有临床研究证据表明，TCE治疗相关ICANS发生率低，以1-2级为主，通过给予支持治疗和/或糖皮质激素治疗，多数情况下ICANS可缓解，因ICANS导致治疗终止的比例低于1%（附表1，http://www.lungca.org/files/2026s61s1.pdf）^[29-31,51]^。提示通过早期识别和及时干预，ICANS亦可获得较好的转归，早期干预能够有效降低其严重程度及病情进展风险。

### 6.3 CRS伴ICANS的处理

**专家共识9：ICANS可能更常发生在CRS之后，但也可能与CRS重叠发生，ICANS和低级别CRS同时发生的情况下，优先处理ICANS；伴发高级别CRS时，除常规ICANS治疗外，推荐使用司妥昔单抗管理CRS（托珠单抗可能增加神经毒性）（证据水平：2类，推荐级别：强）。**

ICANS更常发生于CRS之后，但也可能与CRS同时发生^[54]^。在DeLLphi-304研究^[26]^中，93%的ICANS患者既往发生过CRS。除支持治疗外，糖皮质激素是ICANS管理的主要手段^[51,54]^。鉴于托珠单抗可能存在加重神经毒性的风险，这可能与其阻断IL-6受体后反应性升高的IL-6水平有关^[69]^，当低级别CRS与ICANS同时发生时，应优先处理ICANS。CRS控制不佳且合并ICANS时，可考虑加用抗IL-6抗体司妥昔单抗^[69]^。对于同时发生≥2级CRS和≥2级ICANS的患者，应该考虑收入ICU^[51,54]^。

### 6.4 DLL3-TCE药物因AEs停药后再重启的条件和注意事项

**专家共识10：4级和复发性或持续性的3级CRS或ICANS需要永久停药，其他≤3级的CRS或ICANS，恢复至≤1级后，可在充分评估后考虑重启DLL3-TCE药物治疗。重启TCE药物的治疗，需要关注末次给药的剂量和停药时长，如停药间隔长（2-4周），需重新从低起始剂量开始剂量递增给药和监测，以保证安全性（证据水平：2类，推荐级别：强）。**

AEs恢复后是否重启TCE治疗是临床实践中的常见决策问题。再次使用TCE药物的可行性主要取决于末次AEs的严重程度、患者的一般状况以及停药间隔时间。4级和复发性或持续性的3级CRS或ICANS需要永久停药，其他≤3级的CRS或ICANS，恢复至≤1级后，可在充分评估后考虑重启DLL3-TCE药物治疗，对于血液学毒性、肝脏和感染的AEs转归与恢复治疗可以参考当地临床惯例及时处理（表8）^[51]^。需特别注意的是，若自末次给药以来停药间隔较长，重启TCE治疗时应重新从低剂量开始剂量递增给药，并在治疗过程中参考早期监测方案，以降低再次发生AEs的风险（表9）^[51]^。

**表8:** 塔拉妥单抗AEs恢复后重启治疗的条件

AEs	严重程度	剂量调整
CRS	1-2级	暂停给药直至AEs痊愈
	3级	暂停给药直至AEs痊愈，对于复发性3级AEs，永久停用
	4级	永久停用
ICANS	1-2级	暂停给药直至AEs痊愈
	3级	暂停给药直至AEs痊愈 如果AEs在7天内未改善至≤1级，永久停用 对于复发性3级AEs，永久停用
	4级	永久停用
血细胞减少症	3级中性粒细胞减少症	暂停给药，直至AEs恢复至≤2级 如果3周内AEs未恢复至≤2级，永久停用
	4级中性粒细胞减少症	暂停给药，直至AEs恢复至≤2级 如果1周内AEs未恢复至≤2级，永久停用
	4级中性粒细胞减少症复发	永久停用
	发热性中性粒细胞减少症	暂停给药，直至AEs恢复至≤2级且发热恢复
	血红蛋白<80 g/dL	暂停给药，直至血红蛋白≥80 g/dL
	3或4级血小板计数降低	暂停给药，直至AEs≤2级且无出血证据 如果3周内AEs未恢复至≤2级，永久停用
	4级血小板计数降低复发	永久停用
感染	所有级别	在分步给药阶段的患者暂停给药，直至AEs恢复
	3级	在治疗期间暂停给药，直至AEs恢复至≤1级
	4级	永久停用
肝脏毒性	3级ALT/AST或胆红素升高	暂停给药，直至AEs恢复至≤1级
	4级ALT/AST或胆红素升高	永久停用
	AST/ALT>3×ULN且总胆红素>2×ULN，且无其他原因	永久停用

AEs：不良事件；ALT：丙氨酸氨基转移酶；AST：天门冬氨酸氨基转移酶；ULN：正常值上限。

**表9:** AEs恢复后重启塔拉妥单抗治疗的给药方案

末次塔拉妥单抗给药剂量	距末次塔拉妥单抗给药时间	重启方案*
第1周期第1天，1 mg	2周或更短（≤14天）	给予塔拉妥单抗10 mg，然后按计划剂量和给药方案继续治疗
	超过2周（>14天）	给予塔拉妥单抗1 mg分步剂量；如果可耐受，1周后增加至10 mg； 然后以计划的给药方案重新开始给药
第1周期第8天，10 mg	3周或更短（≤21天）	给予塔拉妥单抗10 mg，然后按计划剂量和给药方案继续治疗
	超过3周（>21天）	给予塔拉妥单抗1 mg分步剂量；如果可耐受，1周后增加至10 mg； 然后以计划的给药方案重新开始给药
第1周期第15天和此后后续周期每2周1次 给予10 mg	4周或更短（≤28天）	给予塔拉妥单抗10 mg，然后按计划剂量和给药方案继续治疗
	超过4周（>28天）	给予塔拉妥单抗1 mg分步剂量；如果可耐受，1周后增加至10 mg； 然后以计划的给药方案重新开始给药

_*_在第1周期第1和8天塔拉妥单抗输注前后给予推荐的伴随用药，并对患者进行相应监测。

## 7 总结与展望

DLL3-TCE药物在SCLC治疗中展现出积极前景，首个DLL3-TCE药物塔拉托单抗在中国获批，多个DLL3-TCE药物进入临床研发阶段，为SCLC治疗带来了新的发展方向。为了更好地指导这一创新药物在临床实践中的使用，专家组制定并撰写了本共识，围绕DLL3-TCE药物用于SCLC治疗的作用机制、临床应用推荐、早期用药与监测及AEs管理等关键临床问题，提出了系统而具体的实践建议。然而，目前仍有很多临床问题有待进一步研究回答，包括：（1）特殊人群用药指导：目前，DLL3-TCE药物的使用数据主要来自于临床研究，对于临床研究没有纳入或纳入少的特殊人群证据有限，如老年患者（≥75岁）、美国东部肿瘤协作组体能状态（Eastern Cooperative Oncology Group performance status, ECOG PS）评分≥2分、肝肾功能不全患者、合并慢性阻塞性肺疾病（chronic obstructive pulmonary disease, COPD）、神经系统疾病患者、伴乙型肝炎病毒（hepatitis B virus, HBV）感染等特殊人群中的疗效、安全性、用药剂量调整及监测要点尚缺乏足够的循证医学证据，未来需要通过更多临床研究和实践经验的积累，为这些人群提供针对性的指导建议，并在后续共识更新中予以补充和完善。（2）优势人群的选择：如患者特征、疗效预测标志物、DLL3表达的疗效预测价值尚存在证据空白，有待更多研究探索，为临床治疗决策提供更多指导依据。（3）联合治疗策略的展望：DLL3-TCE在SCLC中的探索已逐步从后线治疗向一线治疗推进，从单药治疗向联合治疗发展。其中，DLL3-TCE与PD-L1抑制剂和化疗联合用于ES-SCLC一线治疗的研究已进入III期临床试验阶段。此外，多种以DLL3-TCE为基础的新联合策略（如联合ADCs、IL-2抑制剂、放疗等）正在积极探索中，其潜在价值值得期待。（4）中国临床实践操作细则指导：目前DLL3-TCE药物的使用经验主要来自于临床研究，中国临床资源条件下在不同层级医院如何更高效指导DLL3-TCE药物实践操作，有待中国专家在临床实践中积累经验并优化总结。

综上所述，本共识的提出旨在为DLL3-TCE药物在SCLC中的临床应用提供了规范化指导。随着临床研究的深入和临床经验的积累，未来将不断完善相关内容，从而优化SCLC患者的治疗策略。

**共识专家组成员**

**组长：**刘晓晴（解放军总医院第五医学中心），陆舜（上海交通大学附属胸科医院）

**副组长**（按姓氏汉语拼音首字母排序）：范云（浙江省肿瘤医院），黄鼎智（天津市肿瘤医院），刘基巍（大连医科大学附属第一医院），卢铀（四川大学华西医院），邬麟（湖南省肿瘤医院），姚煜（西安交通大学第一附属医院），朱慧（山东第一医科大学附属肿瘤医院）

**执笔**（按姓氏汉语拼音首字母排序）：艾星浩（上海交通大学附属胸科医院），胡洁（上海市老年医学中心），柳影（吉林省肿瘤医院），汪进良（解放军总医院第五医学中心）

**共识组专家**（按姓氏汉语拼音首字母排序）：毕楠（中国医学科学院肿瘤医院），操乐杰（中国科学技术大学附属第一医院/安徽省立医院），陈军（天津医科大学总医院），褚倩（华中科技大学同济医学院附属同济医院），崔久嵬（吉林大学第一医院），董晓荣（华中科技大学同济医学院附属协和医院），方健（北京大学肿瘤医院），高雯（江苏省人民医院），高晓宁（解放军总医院第五医学中心），郭卉（西安交通大学第二附属医院），何雅億（上海市肺科医院），李潞（四川大学华西医院），林根（首都医科大学附属北京胸科医院），刘安文（南昌大学第二附属医院），刘春玲（新疆医科大学附属肿瘤医院），任秀宝（天津医科大学肿瘤医院），宋霞（山西省肿瘤医院），宋勇（南京大学医学院附属金陵医院），涂海燕（广东省人民医院），汪步海（江苏省苏北人民医院），王海永（山东第一医科大学附属肿瘤医院），王佳蕾（复旦大学附属肿瘤医院），王军（河北医科大学第四医院），王立峰（南京大学医学院附属鼓楼医院），王立红（内蒙古医科大学附属医院），王孟昭（北京协和医院），王启鸣（河南省肿瘤医院），徐海鹏（福建省肿瘤医院），杨润祥（云南省肿瘤医院），杨升（福建医科大学附属协和医院），于雁（哈尔滨医科大学附属肿瘤医院），张华（新疆医科大学第一附属医院），张小涛（青岛大学附属青岛市海慈医院），赵艳秋（郑州大学附属肿瘤医院(河南省肿瘤医院)），周承志（广州医科大学附属第一医院），周宁宁（中山大学肿瘤防治中心），朱正飞（复旦大学附属肿瘤医院）
